# Development of a preoperative risk score on admission in surgical intermediate care unit in gastrointestinal cancer surgery

**DOI:** 10.1186/s13741-020-00151-7

**Published:** 2020-08-06

**Authors:** Antero Fernandes, Jéssica Rodrigues, Luís Antunes, Patrícia Lages, Carla Salomé Santos, Daniel Moreira-Gonçalves, Rafael S. Costa, Joaquim Abreu Sousa, Mário Dinis-Ribeiro, Lúcio Lara Santos

**Affiliations:** 1 Experimental Pathology and Therapeutics Group, Portuguese Oncology Institute of Porto FG, EPE (IPO-Porto), Porto, Portugal; 2 Polyvalent Intensive Care Unit, Hospital Garcia de Orta, E.P.E, Almada, Portugal; 3 Epidemiology Service, Portuguese Institute of Oncology of Porto FG, EPE (IPO-Porto), Porto, Portugal; 4 Cancer Epidemiology Group, IPO Porto Research Center (CI-IPOP), Portuguese Oncology Institute of Porto (IPO Porto), Porto, Portugal; 5 Surgical Intermediate Care Unit, Portuguese Institute of Oncology, Porto, Portugal; 6 Research Center in Physical Activity, Health and Leisure (CIAFEL), Faculty of Sport, University of Porto, Porto, Portugal; 7 IDMEC, Instituto Superior Técnico, Universidade de Lisboa, Lisbon, Portugal; 8 REQUIMTE/LAQV, Departamento de Química, Faculdade de Ciências e Tecnologia, Universidade Nova de Lisboa, Caparica, Portugal; 9 Surgical Oncology Department, Portuguese Institute of Oncology of Porto FG, EPE (IPO-Porto), Porto, Portugal; 10 Gastroenterology Department, Portuguese Institute of Oncology of Porto FG, EPE (IPO-Porto), Porto, Portugal

**Keywords:** Oncological digestive surgeries, Postoperative complications, Preoperative risk scoring, Prediction of mortality

## Abstract

**Background:**

Gastrointestinal cancer surgery continues to be a significant cause of postoperative complications and mortality in high-risk patients. It is crucial to identify these patients. Our study aimed to evaluate the accuracy of specific perioperative risk assessment tools to predict postoperative complications, identifying the most informative variables and combining them to test their prediction ability as a new score.

**Methods:**

A prospective cohort study of digestive cancer surgical patients admitted to the surgical intermediate care unit of the Portuguese Oncology Institute of Porto, Portugal was conducted during the period January 2016 to April 2018. Demographic and medical information including sex, age, date from hospital admission, diagnosis, emergency or elective admission, and type of surgery, were collected. We analyzed and compared a set of measurements of surgical risk using the risk assessment instruments P-POSSUM Scoring, ACS NSQIP Surgical Risk Calculator, and ARISCAT Risk Score according to the outcomes classified by the Clavien-Dindo score. According to each risk score system, we studied the expected and observed post-operative complications. We performed a multivariable regression model retaining only the significant variables of these tools (age, gender, physiological P-Possum, and ACS NSQIP serious complication rate) and created a new score (*MyIPOrisk-score*). The predictive ability of each continuous score and the final panel obtained was evaluated using ROC curves and estimating the area under the curve (AUC).

**Results:**

We studied 341 patients. Our results showed that the predictive accuracy and agreement of P-POSSUM Scoring, ACS NSQIP Surgical Risk Calculator, and ARISCAT Risk Score were limited. The *MyIPOrisk-score*, shows to have greater discrimination ability than the one obtained with the other risk tools when evaluated individually (AUC = 0.808; 95% CI: 0.755–0.862). The expected and observed complication rates were similar to the new risk tool as opposed to the other risk calculators.

**Conclusions:**

The feasibility and usefulness of the *MyIPOrisk-score* have been demonstrated for the evaluation of patients undergoing digestive oncologic surgery. However, it requires further testing through a multicenter prospective study to validate the predictive accuracy of the proposed risk score.

## Introduction

Population-based cancer registries worldwide show an increased incidence of gastrointestinal (GI) cancer (Ferlay et al., 2019; Global Burden of Disease Cancer Collaboration, 2017; González & Agudo, 2016). GI cancer includes malignant neoplasms of the esophagus, gallbladder and biliary tract, liver, pancreas, stomach, small intestine, bowel (large intestine or colon and rectum), and anus. Treatment of these tumors mostly involves surgery. Despite the improvements in anesthesia and surgical techniques, GI cancer surgery (GICS) continues to be a major cause of morbidity and mortality (Jhanji et al., 2008; Weiser et al., 2008), contributing to postoperative complications (POC), which in high-risk patients, may be associated with mortality of up to 80% (Mazo et al., 2014; Fernandez-Bustamante et al., 2016). The identification of high-risk patients in the preoperative phase is of crucial importance as it will offer an opportunity to optimize the patient’s status with interventions that contribute to recovery, such as prehabilitation (West et al., 2017).

The American Society of Anesthesiologists Physical Status classification system (ASA PS), P-Possum Score, American College of Surgeons National Surgical Quality Improvement Program (ACS NSQIP), and ARISCAT Risk predictor score for postoperative pulmonary complications are some of the most commonly used perioperative morbidity and mortality risk prediction tools (Hackett et al., 2015; Miskovic & Lumb, 2017; Lubitz et al., 2017; Whiteley et al., 1996). The few prospective studies comparing the accuracy of perioperative risk scoring in GICS and their predictive capacity for mortality and POC provide divergent results, pointing to some limitations in predicting POC. These facts suggest that this area of knowledge is still under-researched (Carvalho-e-Carvalho et al., 2018). Moreover, the lack of consensus on how to define and grade postoperative adverse events has dramatically hampered the evaluation of surgical procedures. To solve this, Clavien-Dindo Classification revealed as an objective and reproducible manner to rank POC complications (Dindo & Clavien, 2004; Chereshneva et al., 2016). Using the classification of surgical complications according to the Clavien-Dindo score, as the outcome, we performed the analysis and comparison of a set of measurements of surgical risk, namely the P-POSSUM Scoring, ACS NSQIP Surgical Risk Calculator, and the ARISCAT Risk Score. The objective was to evaluate their accuracy as perioperative risk assessment instruments in the prediction of postoperative morbidity in GI cancer patients admitted in Surgical Intermediate Care Unit (SICU). The most informative variables from each risk instrument were identified.

## Materials and methods

### Study design and patient population

A cohort study of GI cancer patients admitted to the surgical intermediate care unit (SICU) of the Portuguese Oncology Institute of Porto, Portugal (IPO-Porto) between January 2016 and April 2018 was conducted retrospectively. Throughout this period, we included all consecutive patients aged ≥ 18 years that underwent GI cancer surgery and stayed in the SICU for ≥ 24 h. The IPO-Porto Ethics Committee approved this study. The ethical standards displayed in the 1964 Declaration of Helsinki, and its later amendments were followed. Data were made anonymous for analysis.

### Demographic and medical information

Demographic and medical information including sex, age, date of hospital admission, diagnosis, type of SICU admission: ward-based postoperative complications or elective surgery (elective), and type of surgery were collected and retrospectively entered into an Excel spreadsheet. We also classified patients according to the P-Possum score (since the POSSUM model overestimates the rate of complications in our sample; data not published), ACS NSQIP (without surgeon adjustment of risk), and ARISCAT Risk predictor. Scoring systems and multivariable analysis from the collected data and medical records according to defined criteria were done. Additionally, we studied POC according to the Clavien-Dindo classification.

### Statistical analysis

Continuous variables were described by their median and sample range (min–max). Categorical variables were expressed as actual numbers (*n*) and percentages (%).

To evaluate the association between the occurrence of major complications (Clavien-Dindo ≥ 3) and the potential explanatory variables, we performed a binary logistic regression model. First, considering each variable separately and then making a multivariable model retaining only the significant variables (MyIPOrisk-score). The predictive ability of each continuous score and the final panel obtained was evaluated using receiving operating characteristic (ROC) curves and estimating the area under the curve (AUC). According to the ROC curve, the cutoff was established in order to maximize the Youden’s Index (sensitivity + specificity − 1). Also, the Hosmer–Lemeshow test was used to evaluate the fitted models by comparing the number of predicted complications with the number of observed complications.

We performed a Venn diagram to enhance the relationship between different risk assessment tools in detecting high-risk and low-risk patients as defined by the cutoff value chosen using the criteria explained above. Additionally, we compared the version used in the study with the most recent version announced in the meantime to verify whether the variable serious complications suffered significant changes.

Statistical significance was considered at the level of *P* < 0.05. All statistical analysis was performed using the software R v3.4.4.

## Results

### Description of the GI cancer patients admitted to the SICU

The characteristics of the patients admitted at the SICU are in Table 1. During the study period, a total of 341 patients (59.8% male) that underwent GI cancer surgery (81.5% elective and 18.5% urgent), were admitted in the SICU. Their ages ranged from 22 to 94, with a mean age of 68 years, and approximately 60% of the patients had an ASA score ≥ III.

**Table 1:** Characteristics of the 341 GI cancer patients admitted at the SICU

Characteristics	No. (%)
Age at admission, mean (min–max)	68 (22–94)
Gender	
F	137 (40.2)
M	204 (59.8)
Neoadjuvant chemotherapy	
No	226 (66.3)
Yes	115 (33.7)
Type of surgery (*n*)	
Elective	278 (81.5)
Reoperations	63 (18.5)
ASA	
2	139 (40.9)
3	176 (51.7)
4	25 (7.4)
Surgical category	
Colorectal	103 (30.2)
Esophageal–gastric	60 (17.6)
Hepatic	46 (13.5)
Urgent laparotomies	47 (13.8)
Hyperthermic intraperitoneal chemotherapy (HIPEC)	40 (11.7)
Pancreatic	19 (5.6)
Other	26 (7.6)
Overall complications	181 (53.1)
Clavien-Dindo classification	
Grade I	14 (7.7)
Grade II	82 (45.3)
Grade IIIA	24 (13.3)
Grade IIIB	25 (13.8)
Grade IVA	10 (5.5)
Grade IVB	12 (6.6)
Grade V	14 (7.7)
Mortality	
30 D	12 (3.5)
90 D	22 (6.5)

*F* female, *M* male, *D* days

The distribution of the performed surgeries was as follows: 103 (30.2%) colorectal surgeries, 60 (17.6%) esophageal-gastric surgeries, 46 (13.5%) hepatic surgeries, 47 (13.8%) urgent laparotomies, 40 (11.7%) hyperthermic intraperitoneal chemotherapy (HIPEC), 19 (5.6%) pancreatic surgeries, and 26 (7.6%) other surgeries.

One hundred and fifteen (33.7%) patients also performed chemotherapy in the preoperative period.

Reasons for admission to the SICU were elective surgeries in patients with comorbidities (59, 17.3%), elective complex surgeries (152, 44.6%), reoperations (63, 18.5%), step-down care (47, 13.7%), and postoperative complications (20, 5.9%).

In the universe of 341 patients, the POC rate was 53.1% (181 patients), and its severity according to Clavien-Dindo's classification was Grade I: 14 (7.7%) patients, Grade II: 82 (45.3%) patients, Grade IIIA: 24 (13.3%) patients, Grade IIIB: 25 (13.8%) patients, Grade IVA: 10 (5.5%) patients, Grade IVB: 12 (6.6%) patients, and Grade V: 14 (7.7%) patients. There were 55 deaths with the following distribution: 12 deaths in the first 30 days (11 were surgical complication related), 10 deaths between the 31st and 90th day (3 were surgical complication related), and 33 deaths after the 90th day.

### Analysis post-operative complications by risk score

**Fig. 1:**
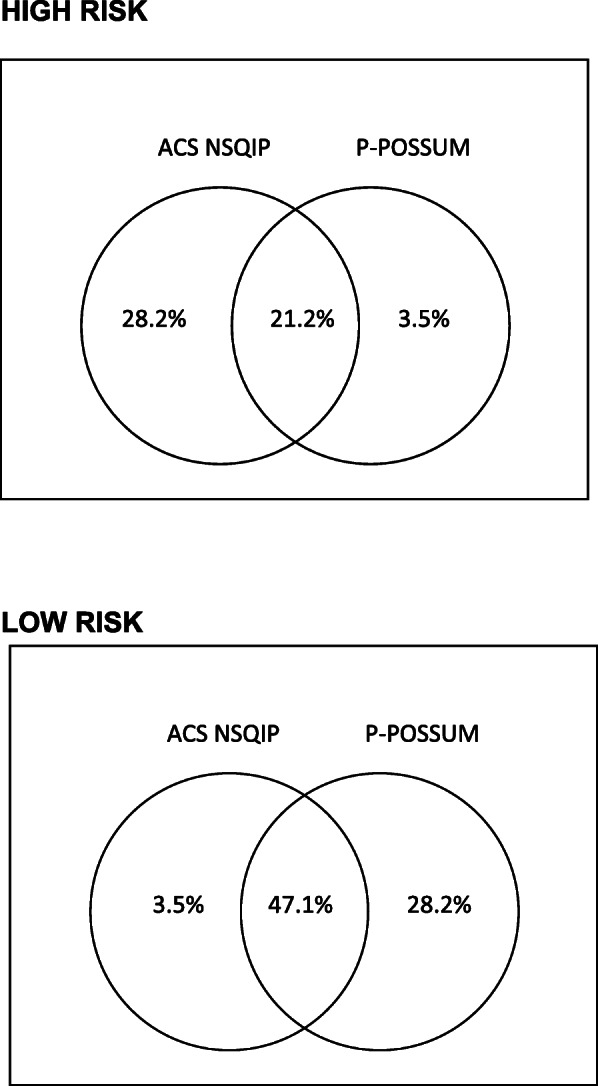
Venn diagram showing the relationship between different risk assessment tools in detecting high-risk and low-risk patients

P-POSSUM predicted a more significant proportion of patients at high risk of morbidity (58.5% vs. 25.7%, respectively) and mortality (12.8% vs. 9.7%, respectively) than the ACS NSQIP Risk Calculator. Venn diagrams in Fig. 1 illustrates the relationship between these two risk score tools in detecting patients at high and low risk of developing complications. As shown, only 21.2% (*n* = 72) of patients were classified as high risk and 47.1% (*n* = 161) as low risk by both tools. Comparing the version used in the study with the most recent version in relation to the variable serious complications did not find significant changes.

Regarding pulmonary complications, ACS NSQIP Risk Calculator predicted that 60.4% of patients could develop pneumonia, and ARISCAT predicted that 67.5% of the patients were at risk of respiratory complications. The number of observed respiratory complications was 38 (11.1%), of which 22 (6.4%) required intensive care support.

### Comparison of the predicted and observed post-operative complications

The Hosmer–Lemeshow goodness of fit test was used to assess the calibration of the risk scores by comparing the observed with anticipated complications by decile of risk (Tables 2 and 3). P-POSSUM showed excellent performance, with an observed and expected complication ratio ranging from 0.76 to 1.23 and an overall good fit (*χ*^2^ = 2.144; *P* = 0.976). On its turn, ACS NSQIP revealed different results. The number of observed complications was less than expected by this tool in low deciles of risk, while the number of expected complications was more significant than the observed ones in higher deciles of risk. Overall, it presented a significant lack of fit (*χ*^2^ = 18.540; *P* = 0.018).

**Table 2:** Hosmer–Lemeshow goodness of fit test for P-Possum for postoperative complications

Deciles of risk (%)	Number of patients	Number of observed complications	Number of expected complications	Mean risk	O:E (95% CI)	X^**2**^HL statistic
0–10	40	4	3.92	0.10	1.02 (0.27–2.61)	0.00
10–20	44	4	5.22	0.12	0.77 (0.21–1.96)	0.32
20–30	24	4	3.26	0.14	1.23 (0.33–3.14)	0.19
30–40	32	6	4.98	0.16	1.20 (0.44–2.62)	0.25
40–50	47	11	9.24	0.20	1.19 (0.59–2.13)	0.42
50–60	27	6	6.30	0.23	0.95 (0.35–2.07)	0.02
60–70	29	6	7.87	0.27	0.76 (0.28–1.66)	0.61
70–80	35	10	11.29	0.32	0.89 (0.42–1.63)	0.22
80–90	33	14	13.80	0.42	1.01 (0.55–1.70)	0.01
90–100	30	20	19.12	0.64	1.05 (0.64–1.62)	0.11
0–100	341	85	85		1.00 (0.80–1.22)	2.14

X^2^HL statistic = 2.144; df = 8; *P* = 0.976

**Table 3:** Hosmer–Lemeshow goodness of fit test for ACS NSQIP for postoperative complications

Deciles of risk (%)	Number of patients	Number of observed complications	Number of expected complications	Mean risk	O:E (95% CI)	X^**2**^HL statistic
0–10	34	1	2.12	0.06	0.47 (0.01–2.63)	0.63
10–20	35	2	3.00	0.09	0.67 (0.07–2.41)	0.37
20–30	33	2	3.62	0.11	0.55 (0.06–1.99)	0.82
30–40	34	8	4.98	0.15	1.61 (0.69–3.17)	2.15
40–50	36	3	6.29	0.17	0.48 (0.10–1.39)	2.09
50–60	32	10	6.71	0.21	1.49 (0.71–2.74)	2.04
60–70	34	15	8.72	0.26	1.72 (0.96–2.84)	6.08
70–80	35	10	11.13	0.32	0.90 (0.43–1.65)	0.17
80–90	33	9	14.69	0.45	0.61 (0.28–1.16)	3.98
90–100	34	25	23.73	0.70	1.05 (0.68–1.56)	0.22
0–100	340	85	85		1.00 (0.80–1.22)	18.54

X^2^HL statistic = 18.540; df = 8; *P* = 0.018

### Multivariable analysis of factors associated with major postoperative complications

Table 4 shows the results of the univariable analysis for major postoperative complications. The significant factors associated with the occurrence of major complications were gender (*P* < 0.001), surgery type (*P* < 0.001), P-POSSUM physiological (*P* < 0.001) and surgical severity (*P* < 0.001), ACS NSQIP (*P* < 0.001), and ARISCAT (*P* = 0.001).

**Table 4:** Association between explanatory variables and major postoperative complications (Clavien-Dindo ≥ 3)

Variable	OR (95% CI)	***P*** value
Age	0.99 (0.97–1.01)	0.240
Gender		
F	1	
M	3.53 (1.97–6.34)	< 0.001
Neoadjuvant chemotherapy		
No	1	
Yes	0.66 (0.38–1.14)	0.135
Surgery type		
Elective	1	
Reoperations	7.47 (4.12–13.53)	< 0.001
ASA		
2	1	
3	1.67 (0.94–2.95)	0.080
4	21.27 (7.22–62.68)	< 0.001
P-Possum		
Physiological	1.11 (1.07–1.15)	< 0.001
Surgical severity	1.20 (1.14–1.27)	< 0.001
ACS NSQIP	1.09 (1.06–1.11)	< 0.001
ARISCAT	1.03 (1.01–1.05)	0.001

*F* female, *M* male

Multivariable logistic regression (Table 5) revealed that occurrence of major complications decreased significantly with age (OR = 0.96; 95%CI: 0.93–0.98), was higher in men (OR = 2.94; 95%CI: 1.52–5.71) and increased with P-Possum (Physiological) score and ACS NSQIP (serious complications) score (OR = 1.08; 95%CI: 1.03–1.12 and OR = 1.06; 95%CI: 1.03–1.09, respectively). We used this model to predict probability of developing postoperative complications and named it as *MyIPOrisk-score*. To dicotomize this score in low/high risk, a cutoff was chosen using the Youden’s index. The cutoff obtained was 23.5 being low risk attributed to patients with a score lower than this value. The equation of predicted postoperative complication (*MyIPOrisk-score*) was as follows:

**Table 5:** Significancy of variables involved in MyIPOriskScore

Variables	OR	95% CI for OR	
		Lower	Upper
Age	0.96	0.93	0.98
Gender (M/F)	2.94	1.52	5.71
PP (physiological)	1.08	1.03	1.12
ACS NSQIP (serious complication)	1.06	1.03	1.09

*F* female, *M* male

Logit (Postoperative complications) = − 2.39 + (− 0.04) × Age + 1.08 if gender is male + 0.07 × P-POSSUM (Physiological) + 0.06 × ACS NSQIP (Serious complications)

**Fig. 2:**
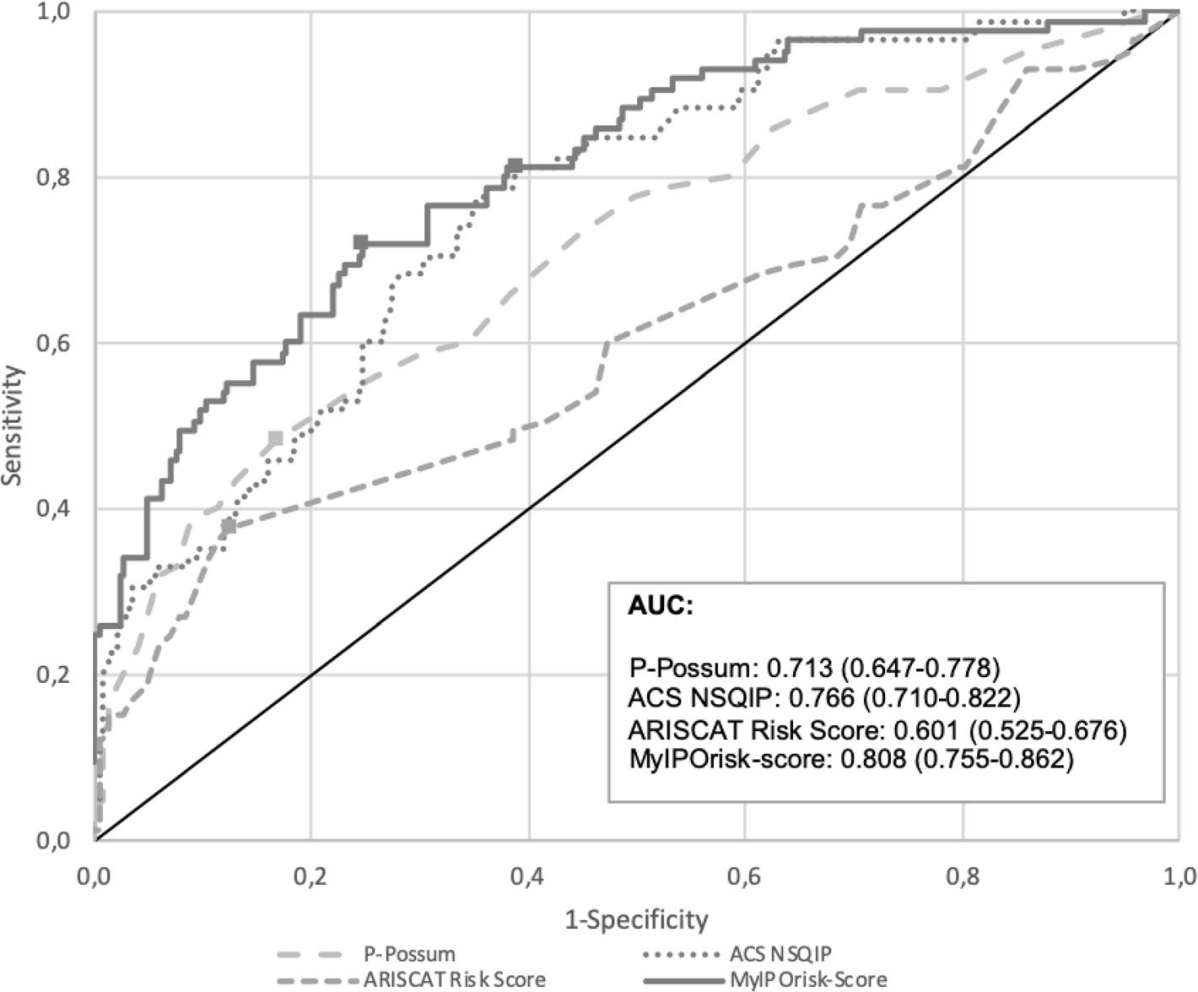
ROC curve for the P-Possum, ACS NSQIP, ARISCAT, and MyIPOrisk-score scoring systems for predicting the rate of postoperative morbidity (i.e., complications according to the Clavien-Dindo classification) in patients undergoing GICS. *ROC* receiver operating characteristic, *AUC* area under the curve

*MyIPOrisk-score* showed no significant lack of fit (Table 6) (*χ*^2^ = 4.44; *P* = 0.815). The discriminatory ability of the *MyIPOrisk-score* obtained with the final model (AUC = 0.808; 95%CI: 0.755–0.862) was significantly higher than the ability of each score individually (*MyIPOrisk-score* vs. ACS NSQIP: *P* = 0.047; *MyIPOrisk-score* vs. P-Possum: *P* = 0.028) (Fig. 2).

**Table 6:** Hosmer–Lemeshow goodness of fit test for MyIPOriskScore for postoperative complications

Deciles of risk (%)	Number of patients	Number of observed complications	Number of expected complications	Mean risk	O:E (95% CI)	X^**2**^HL statistic
0–10	34	2	1.15	0.03	1.75 (0.20–6.30)	0.66
10–20	34	0	2.07	0.06	0.00 (-)	2.20
20–30	34	3	3.06	0.09	0.98 (0.20–2.86)	0.00
30–40	34	4	4.01	0.12	1.00 (0.27–2.55)	0.00
40–50	34	7	5.25	0.15	1.33 (0.53–2.75)	0.69
50–60	34	8	6.34	0.19	1.26 (0.54–2.49)	0.53
60–70	34	7	8.40	0.25	0.83 (0.33–1.72)	0.31
70–80	34	11	10.91	0.32	1.01 (0.50–1.80)	0.00
80–90	34	16	16.42	0.48	0.97 (0.56–1.58)	0.02
90–100	34	27	27.39	0.81	0.99 (0.65–1.43)	0.03
0–100	340	85	85		1.00 (0.80–1.22)	4.44

X^2^HL statistic = 4.440; df = 8; *P* = 0.815

## Discussion

In this study, we analyzed and compared the surgical risk obtained by P-POSSUM Scoring, ACS NSQIP Surgical Risk Calculator, and ARISCAT Risk Score according to the outcomes classified by the Clavien-Dindo score. We aimed to evaluate their accuracy as perioperative risk assessment instruments to predict postoperative morbidity and to identify the most informative variables. Overall, our data suggest that (i) these instruments have a poor predictive performance for POC; (ii) P-POSSUM and ACS NSQIP Risk Calculator have poor agreement for the identification of patients at high risk for morbidity; and (iii) combining the most informative variables of current risk models was superior in predicting POC than each score individually.

The perioperative period is the perfect opportunity to identify patients with increased risk profile for shared and individualized decision-making and preoperative optimization (e.g., prehabilitation) with the ultimate goal of providing better outcomes (Hijazi et al., 2017). For that purpose, several classical risk prediction models (e.g., P-POSSUM Scoring, ACS NSQIP Surgical Risk Calculator, and the ARISCAT Risk Score) were developed and prospectively validated and are currently used worldwide (Huang et al., 2015; Lee et al., 2012; Copeland et al., 1991; Haga et al., 1999; Miki et al., 2014; Kim et al., 2008). However, a significant variation in terms of the diagnostic accuracy of these models has been reported in various surgical specialties, rising doubts about their generalization (Kumagai et al., 2014; Yu et al., 2016; SAH et al., n.d.). We observed a poor accuracy and agreement (below 50%) between the studied models in our cohort of GI cancer patients admitted to the SICU, cautioning us to their routine use to assess preoperative risk for POC and support precision management decisions.

To overcome this limitation, we performed this training set study and identified the most informative variables from current risk models assessed in our study, with major complications (Clavien-Dindo ≥ 3) as the outcome measure of reference. Binary logistic regression identified that the occurrence of major complications decreased significantly with age (OR = 0.96; 95%CI: 0.93–0.98), was higher in men (OR = 2.94; 95%CI: 1.52–5.71) and increased with P-Possum (Physiological) score and serious complications ACS score (OR = 1.08; 95%CI: 1.03–1.12 and OR = 1.06; 95%CI: 1.03–1.09, respectively). The decrease of risk with age is probably explained by the avoidance of complex surgical procedures performed in older patients. When considered alone, the ARISCAT score was also associated with the occurrence of major complications but lost significance after adjusting for the other variables. Our results are in agreement with Scott S et al. (Scott et al., 2014), who found that the Physiological score of POSSUM and P-POSSUM had higher discrimination than the Operative score in predicting postoperative mortality at a critical care setting. We did not find significant POC variation according to age and gender, although there are references in the literature about a relative preponderance in young patients undergoing surgery for GI cancer, probably due to more extensive operations to which they are submitted. As for gender discrimination, it seems to depend more on the type of tumor involved (Alves et al., 2002; Knoferl et al., 2002; Schroder et al., 1998).

Choi M et al., when testing the potential feasibility of the ACS NSQIP Surgical Risk Calculator for predicting long-term cancer outcomes in patients with resected pancreatic head cancer, found that the serious complication rate parameter calculated with this risk assessment instrument was the most informative (Choi et al., 2019).

Based on the informative variables of current risk models, we constructed a model with a greater accuracy to predict complications in the postoperative period in GI cancer patients in need of surgery, that we named *MyIPOrisk-score.* The discrimination ability of the *MyIPOrisk-score* obtained with the final model (AUC = 0.808; 95%CI: 0.755–0.862) was significantly higher than each score individually (*MyIPOrisk-score* vs ACS NSQIP: *P* = 0.047; *MyIPOrisk-score* vs P-Possum: *P* = 0.028). These results are very similar to those recently published by Bihorac A et al. (Bihorac et al., 2019) that developed and validated, in a cohort of 51,457 surgical patients undergoing major inpatient surgery, an automated analytics framework for a preoperative risk algorithm to forecast patient-level probabilistic risk scores for 8 major postoperative complications (acute kidney injury, sepsis, venous thromboembolism, intensive care unit admission > 48 h, mechanical ventilation > 48 h, wound, neurologic, and cardiovascular complications) and death up to 24 months after surgery. This model calculates probabilistic risk scores for 8 postoperative complications with AUC values ranging between 0.82 and 0.94 (99% confidence intervals (CIs) 0.81–0.94). (Schroder et al., 1998) Importantly, the Hosmer–Lemeshow equation revealed that *MyIPOrisk-score* presented the best association between the number of observed complications and the number of expected complications.

Our study is not free of limitations. It was a single-center retrospective study, and some of the data were collected from medical records, which could be a source of bias due to the need of interpreting data. NSQIP may change their model discrimination or calibration. However, our results did not present any quality change when we used the latest versions of this score and compared with the previous (the rate of serious complications is stable). Although *MyIPOrisk-score* needs other scores to obtain a prediction, these are available for everyone. The feasibility of the *MyIPOrisk-score* now requires further testing through multicenter prospective studies to validate the predictive accuracy of the proposed risk score.

The main interest in the use of this score is to identify more accurately patients with high risk of having postoperative complications so that they can be subjected to a prehabilitation program in order to optimize their performance in preoperative time and a postoperative care in the SICU.

## Conclusion

Based on the most informative variables of current risk models, we developed a surgical risk score instrument that showed greater performance in predicting risk of surgical complications in GI cancer surgeries. However, it will be necessary to evaluate its performance using a validation set.

## Data Availability

The datasets generated and/or analyzed during the current study are available in the IPO-PORTO repository. The datasets used and/or analyzed during the current study are available from the corresponding author on reasonable request.

## Abbreviations

ACS NSQIP: American College of Surgeons National Surgical Quality Improvement Program
AUC: Area under the curve
ASA PS: Anesthesiologists Physical Status classification system
ARISCAT: Assess Respiratory Risk in Surgical Patients in Catalonia
GI: Gastrointestinal
GICS: GI cancer surgery
IPO-Porto: Portuguese Oncology Institute of Porto, Portugal
MyIPOrisk-score: Our new risk score
POC: Postoperative complications
P-Possum: Physiological and Operative Severity Score for the enumeration of Mortality and morbidity (POSSUM) and Portsmouth-POSSUM *scores*
ROC curves: Receiver operating characteristic curves
SICU: Surgical Intermediate Care Unit
